# Ophthalmic and Cutaneous Manifestation of Xeroderma Pigmentosum in a 21-Year-Old Man: A Case Report

**DOI:** 10.7759/cureus.85947

**Published:** 2025-06-13

**Authors:** Brian A Moreno, Moises Lutwak, Stanley Skopit

**Affiliations:** 1 Dermatology, Lake Erie College of Osteopathic Medicine, Bradenton, USA; 2 Dermatology, Larkin Community Hospital, South Miami, USA

**Keywords:** academic dermatology, clinical dermatology, complex dermatology, dermatology and dermatologic surgery, dermatology care, dermatology clinics, dermatology oncology, general dermatology, search in dermatology, skin disease/dermatology

## Abstract

Xeroderma pigmentosum (XP) is a rare autosomal recessive disorder characterized by a defect in DNA repair, leading to marked sensitivity to ultraviolet (UV) light, an increased risk of cutaneous malignancies, and frequent ophthalmic complications. We present a 21-year-old man with a history of bilateral ocular melanomas and evolving cutaneous lesions suspicious for malignancy. This report highlights the clinical features, histopathologic considerations, and multidisciplinary management challenges in XP, underscoring the importance of vigilant surveillance, early intervention, and interdisciplinary care.

## Introduction

Xeroderma pigmentosum (XP) is a rare genetic disorder with an estimated incidence of 1 per 1,000,000 in North America and Europe. It is characterized by nucleotide excision repair defects or, in the XP-variant (XP-V) type, a defect in the DNA polymerase η-mediated bypass repair process [1,2]. Patients exhibit increased sensitivity to UV radiation, leading to early onset freckling, multiple cutaneous malignancies, ocular surface disease, and, in approximately 20-30% of cases, progressive neurological abnormalities [1,3,4]. Ocular involvement is common and may include eyelid tumors, conjunctival injection, pterygia, and corneal opacities, as well as intraocular manifestations such as retinal pigmentary changes and optic atrophy [5,6].

We report a case of a young adult man with XP presenting with bilateral ocular melanomas in his past medical history, ongoing skin lesions suspicious for malignancy, and evolving cutaneous manifestations. This case underscores the complexity of diagnosing and managing XP-related malignancies and ocular complications.

## Case presentation

A 21-year-old man with a known history of XP presented for a full-body skin examination. The patient had previously undergone ocular surgery for malignant melanoma in both eyes and nasal surgery related to melanoma management, all without complications. He also required enteral nutrition support via a feeding tube, possibly reflecting neurologic involvement sometimes seen in XP. Family history was negative for melanoma, consistent with the autosomal recessive inheritance pattern of XP rather than familial melanoma syndromes.

**Figure 1:**
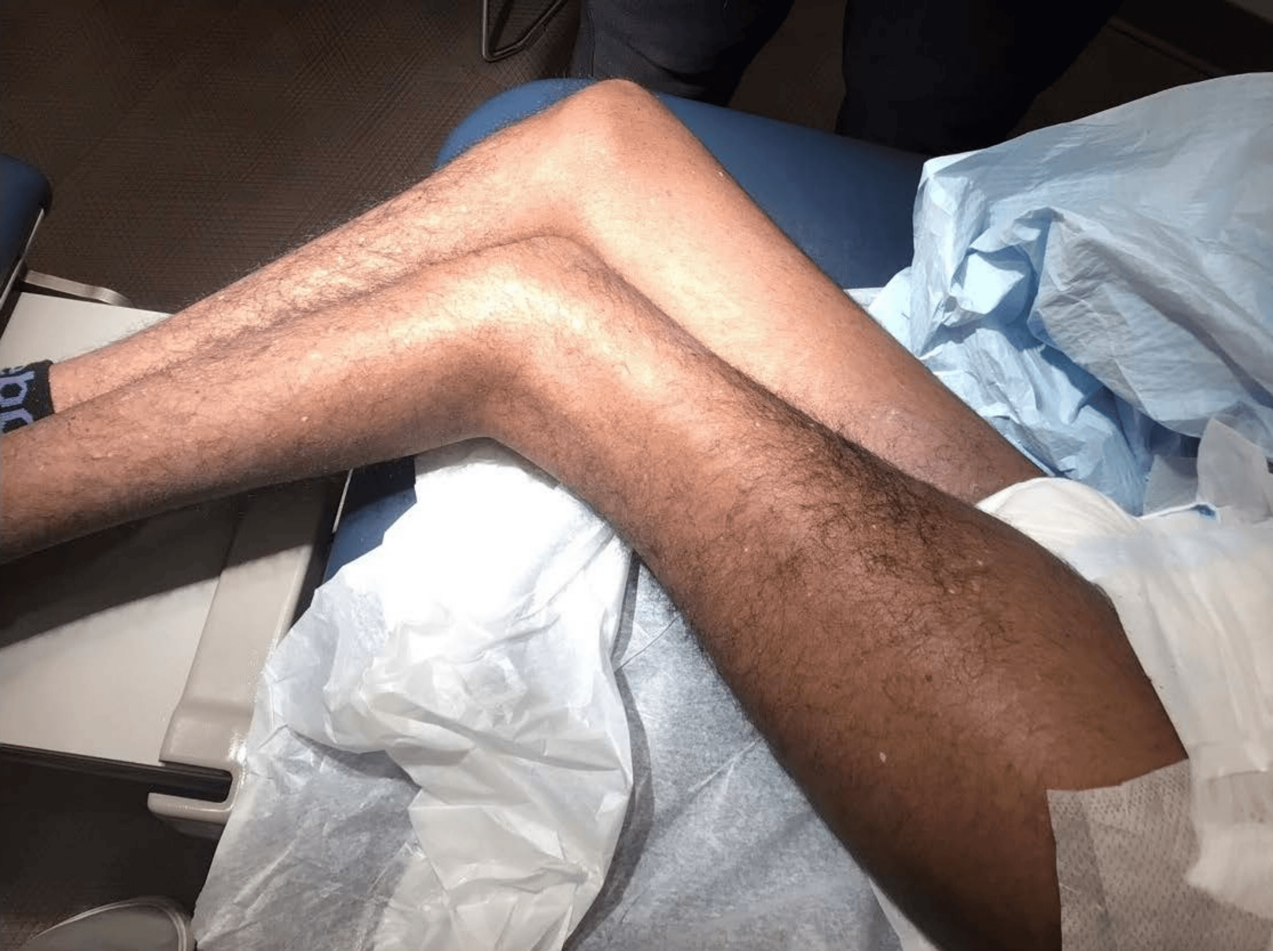
Diffuse lentigines, hypopigmented macules, and actinic keratoses on the lower extremities.

**Figure 2:**
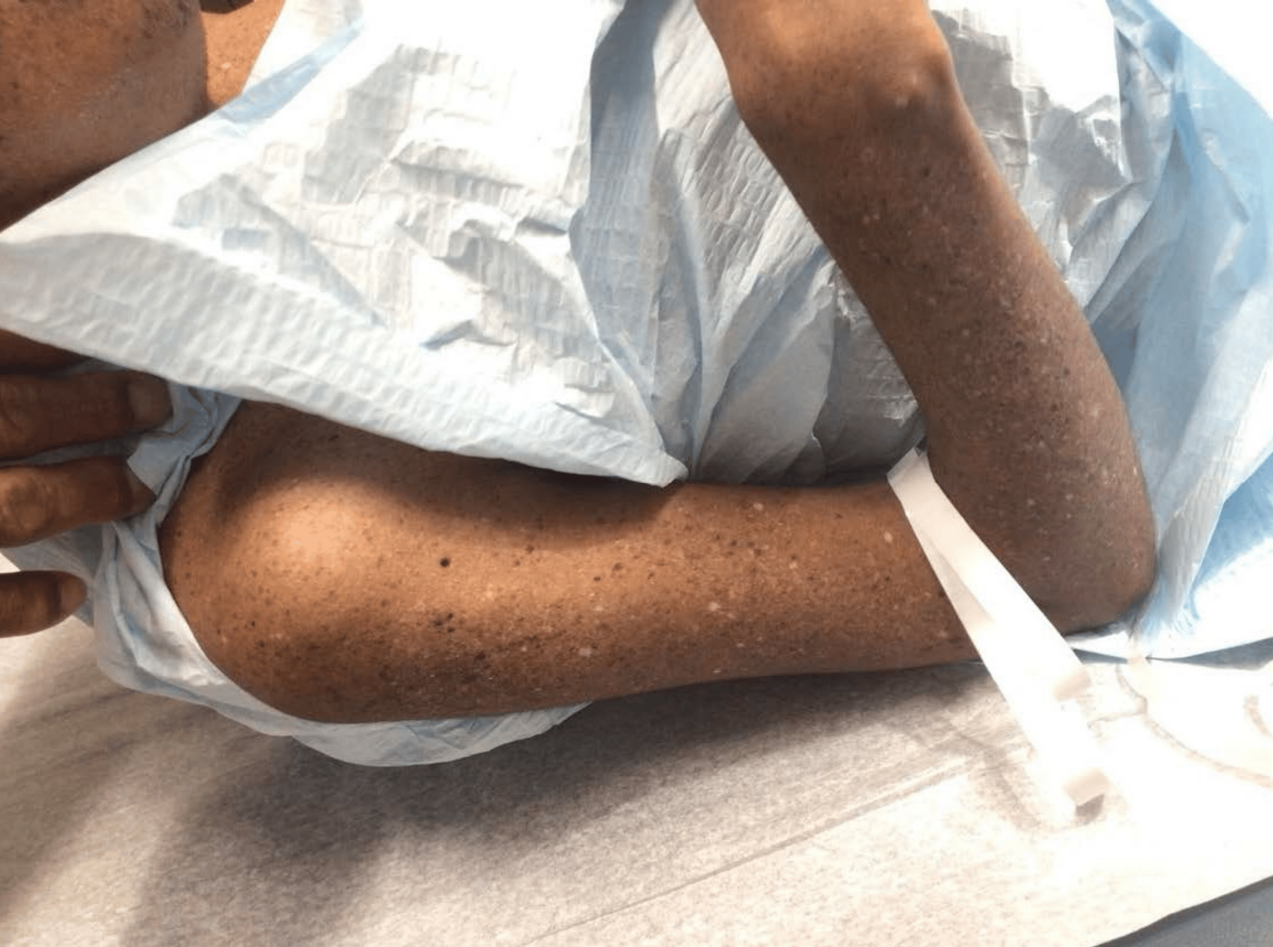
Diffuse lentigines, hypopigmented macules, and actinic keratoses on the right arm.

**Figure 3:**
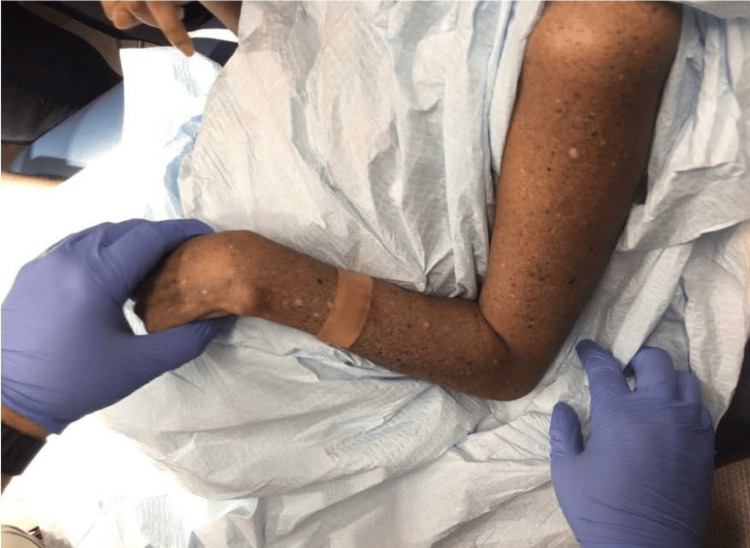
Diffuse lentigines, hypopigmented macules, and actinic keratoses on the left arm.

**Figure 4:**
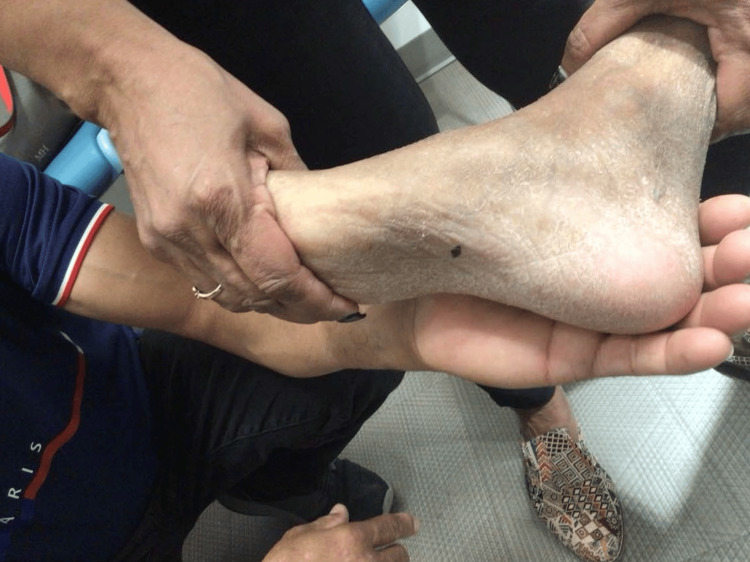
Lesion on the plantar aspect of the foot, suspicious for dysplastic nevus or early melanoma.

On examination, diffuse lentigines and hypopigmented macules were noted on sun-exposed areas (Figures 1-3). A suspicious pigmented scaly papule was identified on the left distal dorsal forearm (Figure 3). Differential diagnoses included irritated seborrheic keratosis, squamous cell carcinoma (SCC), basal cell carcinoma (BCC), or melanoma. Another lesion on the plantar aspect of the foot appeared suspicious for dysplastic nevus or early melanoma (Figure 4). Although the patient’s mother deferred immediate biopsy of the plantar lesion, a shave biopsy of the dorsal forearm papule was performed and sent for histopathological evaluation. Histology revealed a benign melanocytic nevus, predominantly intradermal type, with associated inflammation.

**Figure 5:**
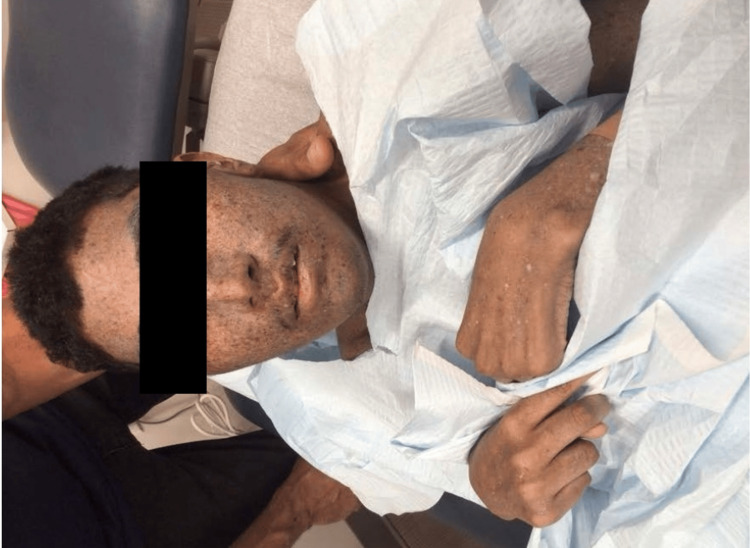
Perioral dermatitis around the mouth.

Additionally, the patient displayed perioral dermatitis (Figure 5) and actinic keratoses on the extremities (Figures 1-3), both commonly associated with chronic UV exposure [7]. Onychomycosis was also suspected, which can be incidental in XP patients but underscores their heightened susceptibility to dermatologic conditions.

Ocular assessment was significant for a history of bilateral malignant melanoma of the eyes. Ophthalmic manifestations of XP can include dry eye, exposure keratopathy, pterygia, pinguecula, and conjunctival or corneal neoplasms [5,6]. In this patient, previous ocular involvement required surgical intervention and close ophthalmology follow-up. At present, the ocular exam revealed residual dryness and mild photoaging changes.

## Discussion

XP is caused by defects in DNA repair, specifically nucleotide excision repair genes (XPA-XPG) or POLH (XP-variant), resulting in inefficient repair of UV-induced DNA lesions [1,2]. The hallmark feature is the early onset of cutaneous and ocular changes upon minimal UV exposure. Patients often develop multiple cutaneous malignancies at a young age, including BCC, SCC, and melanoma [2,4]. Ocular involvement predominantly affects the anterior segment and periocular skin, but involvement of deeper ocular structures, including the retina and optic nerve, has been reported [5].

Our patient’s complex history of bilateral ocular melanoma aligns with literature describing ocular surface and intraocular involvement in XP [5,6]. In-depth histopathological studies have documented corneal pannus, conjunctival tumors, retinal pigmentary changes, and optic atrophy in XP patients [5]. Similarly, systemic neurologic manifestations can occur due to persistent DNA damage affecting the central nervous system [1,4]. Although neurological status was not detailed in this case, the need for a feeding tube may suggest neurologic involvement.

Management requires a multidisciplinary approach with dermatology, ophthalmology, oncology, neurology, and genetics. Strict photoprotection measures, including sun avoidance, protective clothing, and regular use of broad-spectrum sunscreen, are paramount. Early surgical intervention for suspicious lesions is critical. In our case, a shave biopsy was performed to establish a definitive diagnosis. If confirmed malignant, local excision is often the treatment of choice, and Mohs micrographic surgery may be considered for periocular tumors [5,7]. Patients benefit from regular skin and eye examinations, prompt identification and excision of neoplasms, and genetic counseling [8].

## Conclusions

This case of a 21-year-old man with XP and a history of bilateral ocular melanoma, as well as evolving suspicious cutaneous lesions, highlights the disease’s complexities and the critical need for early detection and comprehensive care. Given XP’s rarity and variable phenotype, clinicians must remain vigilant. Multidisciplinary management, photoprotection, regular surveillance, and early intervention are central to improving outcomes in XP patients.
